# The efficacy of insecticide-treated window screens and eaves against *Anopheles* mosquitoes: a scoping review

**DOI:** 10.1186/s12936-021-03920-x

**Published:** 2021-09-29

**Authors:** Beverly I. Anaele, Karan Varshney, Francis S. O. Ugwu, Rosemary Frasso

**Affiliations:** 1 College of Population Health, Thomas Jefferson University, Philadelphia, PA USA; 2 School of Medicine, Deakin University, Geelong, VIC Australia; 3 South East Zonal Biotechnology Centre and Department of Zoology and Environmental Biology, University of Nigeria, Nsukka, Nigeria

**Keywords:** Malaria, Window screen, Eave, Insecticide, Scoping review

## Abstract

**Background:**

Female mosquitoes serve as vectors for a host of illnesses, including malaria, spread by the *Plasmodium* parasite. Despite monumental strides to reduce this disease burden through tools such as bed nets, the rate of these gains is slowing. Ongoing disruptions related to the COVID-19 pandemic may also negatively impact gains. The following scoping review was conducted to examine novel means of reversing this trend by exploring the efficacy of insecticide-treated window screens or eaves to reduce *Anopheles* mosquito bites, mosquito house entry, and density.

**Methods:**

Two reviewers independently searched PubMed, Scopus, and ProQuest databases on 10 July, 2020 for peer-reviewed studies using insecticide-treated screens or eaves in malaria-endemic countries. These articles were published in English between the years 2000–2020. Upon collection, the reports were stratified into categories of biting incidence and protective efficacy, mosquito entry and density, and mosquito mortality.

**Results:**

Thirteen out of 2180 articles were included in the final review. Eaves treated with beta-cyfluthrin, transfluthrin or bendiocarb insecticides were found to produce vast drops in blood-feeding, biting or mosquito prevalence. Transfluthrin-treated eaves were reported to have greater efficacy at reducing mosquito biting: Rates dropped by 100% both indoors and outdoors under eave ribbon treatments of 0.2% transfluthrin (95% CI 0.00–0.00; p < 0.001). Additionally, co-treating window screens and eaves with polyacrylate-binding agents and with pirimiphos-methyl has been shown to retain insecticidal potency after several washes, with a mosquito mortality rate of 94% after 20 washes (95% CI 0.74–0.98; p < 0.001).

**Conclusions:**

The results from this scoping review suggest that there is value in implementing treated eave tubes or window screens. More data are needed to study the longevity of screens and household attitudes toward these interventions.

## Background

In 2019, the World Health Organization (WHO) reported a total of 229 million cases of malaria [1]. Sub-Saharan Africa carries the majority of the malaria burden, equating to 94% of worldwide cases. Several reasons for this disproportionality exist, one of which lies in housing infrastructure [2]. As evidence of this, a study indicated that on average malaria doubled among children with the lowest socio-economic status (SES) when measured in household wealth, parental education level and job status relative to children in the same community with the highest SES [3]. The researchers hypothesized that this discrepancy in malaria contraction may have derived from differences in access to healthcare, knowledge of treatment opportunities, food security, and housing quality [3].

With improved housing comes a reduced risk of contracting malaria due to lowered exposure to mosquitoes [2, 4, 5]. A 2001–2017 cross-sectional analysis using data from 33 African countries found that improved housing lowered the risk of child mortality from malaria by 12–18% [4]. Openings in walls and other points of entry, once closed, can bar vectors from biting indoors during their active hours. Tools such as insecticide-treated window screens and eaves are designed to do just that, along with the added community effect of reducing mosquito populations due to the insecticide treatment. However, these devices are not as widely prioritized in the fight against malaria compared to bed net programmes or indoor residual spraying [6]. More concrete research displaying the benefits of window screens and eaves is required [6]. Added research will help to motivate all critical stakeholders, such as policymakers, to invest in said tools. Addressing housing quality could help to alleviate other issues of poverty by identifying household needs and concerns.

The following review addresses the call for more research by synthesizing and summarizing current scientific knowledge of housing interventions to inform policy. More specifically, this scoping review provides a detailed account of the literature, assessing whether chemical insecticide-treated window screens and eaves are capable of reducing malaria incidence, prevalence, and mosquito biting densities within malaria-endemic countries.

## Methods

### Search strategy

On 10 July, 2020, the research team performed a literature search exploring PubMed, Scopus, and ProQuest databases using the terms found in Table 1. These search terms were selected through collaboration with a research librarian and incorporated medical subject headings and keywords. Both categories pertained to malaria-endemic regions, insecticides or pyrethroids, window screens, and eaves. To address outcomes, search terms on epidemiology, mosquito bites, disease prevalence, and incidence were also included.

**Table.1:** Search strategies and databases performed on July 10, 2020

Database	Search	Hits
PubMed	Sub-Saharan Africa OR “Africa South of the Sahara”[Mesh] OR Southeast Asia OR “Asia, Southeastern”[Mesh] OR Eastern Mediterranean OR “Morocco”[Mesh] OR “Cyprus”[Mesh] OR Western Pacific OR “Ecuador”[Mesh] OR “Peru”[Mesh] OR “Samoa”[Mesh] OR “Independent State of Samoa”[Mesh] OR “Palau”[Mesh] OR South America OR “South America”[Mesh] AND Malaria OR “Malaria”[MeSH Terms] OR “Plasmodium”[Mesh] AND insecticide OR insects OR Insecticidal OR “Pyrethrins”[Mesh] OR pyrethroid OR “Mosquito Control”[Mesh] OR Insecticide-treated AND windows OR window OR screens OR screen OR eaves OR eave AND Mosquito bites OR Mosquito bite OR “culicidae”[MeSH Terms] OR mosquito OR “bites and stings”[MeSH Terms] OR bites stings OR bites OR “Malaria/epidemiology”[Mesh] OR “Malaria/prevention and control”[Mesh]	637
ProQuest	(Sub-Saharan Africa OR Africa OR Southeast Asia OR SOUTHEAST ASIA OR Mediterranean OR Mediterranean Area OR Morocco OR Cyprus OR Western Pacific OR Ecuador OR Peru OR Samoa OR Palau OR South America OR SOUTH AMERICA) AND (malaria OR plasmodium OR plasmodium berghei OR plasmodium chabaudi OR plasmodium cynomolgi OR plasmodium falciparum OR plasmodium falciparum (malaria parasite) OR plasmodium gallinaceum OR plasmodium knowlesi OR plasmodium malariae OR plasmodium ovale OR plasmodium relictum OR plasmodium vinckei OR plasmodium vivax OR plasmodium yoelii) AND (Insecticide OR insecticides OR pyrethrins OR pyrethroid OR mosquito control) AND (window OR windows OR window screens OR screen doors OR eaves OR eaves.) AND (bites OR bites & stings OR bites, human)	1018
Scopus	TITLE-ABS-KEY sub-saharan AND africa OR TITLE-ABS-KEY africa AND south AND of AND the AND sahara OR TITLE-ABS-KEY southeast AND asia OR TITLE-ABS-KEY eastern AND mediterranean OR TITLE-ABS-KEY morocco OR TITLE-ABS-KEY southeastern AND asia OR TITLE-ABS-KEY cyprus OR TITLE-ABS-KEY western AND pacific OR TITLE-ABS-KEY ecuador OR TITLE-ABS-KEY peru OR TITLE-ABS-KEY samoa OR TITLE-ABS-KEY independent AND state AND of AND samoa OR TITLE-ABS-KEY palau OR TITLE-ABS-KEY south AND america AND TITLE-ABS-KEY malaria OR TITLE-ABS-KEY plasmodium AND TITLE-ABS-KEY insecticide OR TITLE-ABS-KEY insects OR TITLE-ABS-KEY insecticidal OR TITLE-ABS-KEY pyrethrins OR TITLE-ABS-KEY pyrethroid OR TITLE-ABS-KEY mosquito AND control OR TITLE-ABS-KEY insecticide-treated AND TITLE-ABS-KEY windows OR TITLE-ABS-KEY window OR TITLE-ABS-KEY screens OR TITLE-ABS-KEY screen OR TITLE-ABS-KEY eaves OR TITLE-ABS-KEY eave OR TITLE-ABS-KEY net OR TITLE-ABS-KEY nets OR TITLE-ABS-KEY bednet OR TITLE-ABS-KEY bednets OR TITLE-ABS-KEY bed AND net OR TITLE-ABS-KEY bed AND nets AND TITLE-ABS-KEY epidemiology OR TITLE-ABS-KEY mosquito AND bites OR TITLE-ABS-KEY mosquito AND bite OR TITLE-ABS-KEY culicidae OR TITLE-ABS-KEY mosquito OR TITLE-ABS-KEY stings OR TITLE-ABS-KEY bites OR TITLE-ABS-KEY prevalence OR TITLE-ABS-KEY incidence	525

### Inclusion and exclusion criteria

The research team incorporated guidelines from the Preferred Reporting Items for Systematic Reviews and Meta-Analyses (PRISMA) study selection process [7]. The inclusion criteria comprised: (1) data reported from malaria-endemic countries in the WHO regions of sub-Saharan Africa, Southeast Asia, the Eastern Mediterranean, the Western Pacific, and South America; (2) studies reported in English; (3) interventions investigating *Anopheles* mosquitoes using insecticide-treated netting for window screens and eaves; (4) studies that used empirical methods, meaning the collection and analysis of data using the scientific method; and (5) studies that were published in the years 2000–2020. Articles that investigated bed nets or curtains alone were excluded from the study.

### Data extraction

Data were collected in RefWorks Legacy, and each article was independently assessed by two reviewers (BIA and KV). Data from each study were qualitatively synthesized into categories of biting incidence and protective efficacy, mosquito entry and density, mosquito mortality, and household member attitudes toward each intervention. The Critical Appraisal Skills Programme Checklist (CASP) for case–control studies was used to evaluate the quality of these reports, where groups exposed to the insecticide were taken as cases and non-exposed groups were categorized as controls [8]. Randomized controlled trials (RCTs) were appraised with the CASP for RCT studies [9]. Reviewers discussed and resolved any discrepancies.

## Results

**Fig. 1:**
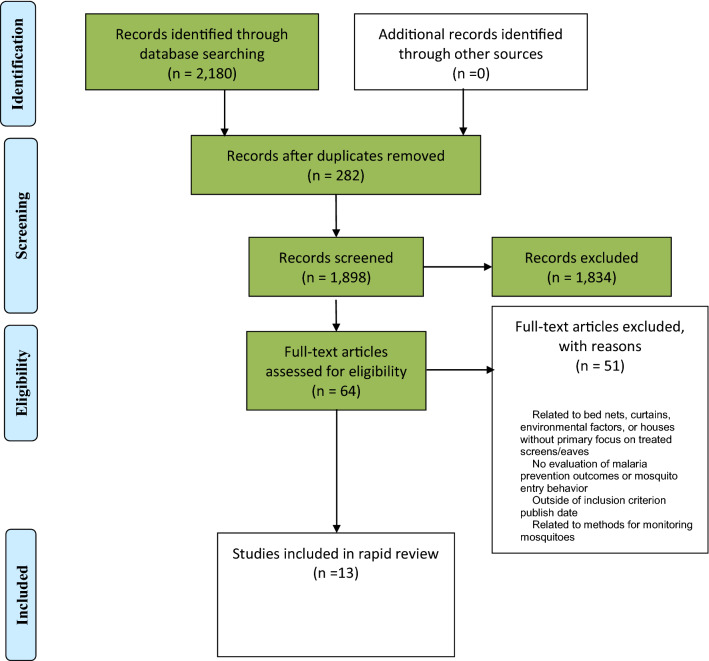
Flowchart of study selection process

Out of 2180 results, 282 duplicates were removed and 1834 studies excluded after assessing their titles and abstracts. Thirteen final articles were identified out of the remaining 64 studies that met all the inclusion criteria (Fig. 1) [10–22]. These 13 articles assessed interventions based on their overall effect on biting incidence, protective efficacy, mosquito entry and density, mosquito mortality, and participant attitudes toward the mosquito control interventions. All articles were of high quality based on their appropriate appraisal checklist (Table 2).

**Table.2:**
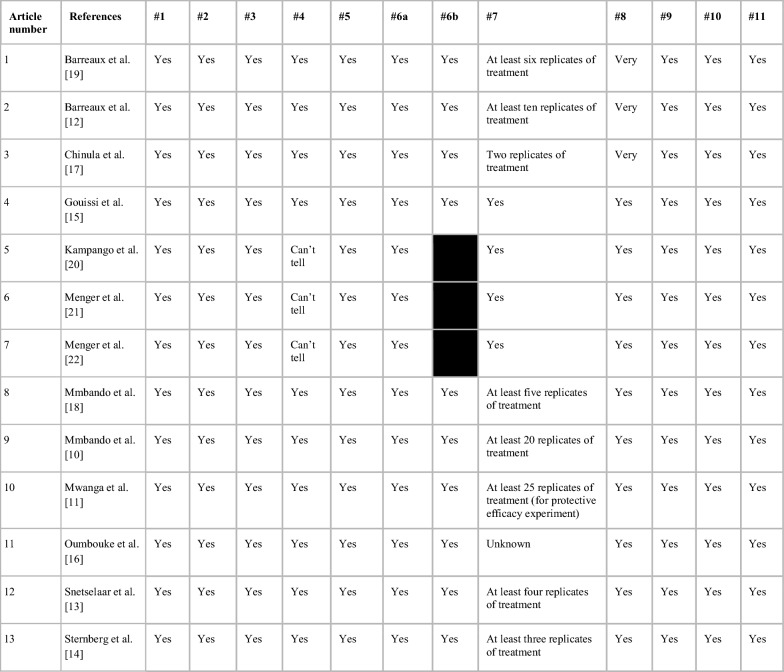
Critical appraisal checklist

Case–control (articles 1–4; 8–13)

#1. Did the study address a clearly focused issue?

#2. Did the authors use an appropriate method to answer their question?

#3. Were the cases recruited in an acceptable way?

#4. Were the controls selected in an acceptable way?

#5. Was the exposure accurately measured to minimise bias?

#6a. Aside from the experimental intervention, were the groups treated equally?

#6b. Have the authors taken account of the potential confounding factors in the design and/or in their analysis?

#7. How large was the treatment effect?

#8. How precise was the estimate of the treatment effect?

#9. Do you believe the results?

#10. Can the results be applied to the local population?

#11. Do the results of this study fit with other available evidence?

Randomized controlled trial (articles 5–7):

#1. Did the study address a clearly focused research question?

#2. Was the assignment of participants to interventions randomised?

#3. Were all participants who entered the study accounted for at its conclusion?

#4. Were the participants ‘blind’ to the intervention they were given? Were the investigators ‘blind’ to the intervention they were giving to participants? Were the people assessing/analysing outcome/s ‘blinded’?

#5. Were the study groups similar at the start of the randomised controlled trial?

#6. Apart from the experimental intervention, did each study group receive the same level of care (that is, were they treated equally)?

#7. Were the effects of intervention reported comprehensively?

#8. Was the precision of the estimate of the intervention or treatment effect reported?

#9. Do the benefits of the experimental intervention outweigh the harms and costs?

#10. Can the results be applied to your local population/in your context?

#11. Would the experimental intervention provide greater value to the people in your care than any of the existing interventions?

The black shaded areas for these articles indicate the absence of an additional critical appraisal question

### Characteristics of studies

Population demographics of all articles varied widely, particularly in the *Anopheles* species that researchers studied (Table 3). Three interventions utilized RCT study designs and the remaining 10 used case–control release-recapture, wild mosquito recruitment, and/or experimental hut studies (Table 3). Studies using experimental huts first reconstructed West African-style homes. These homes were modified to include multiple eave tubes at eave level. Other reports identified as village studies, as they incorporated housing units already present in a community. To collect mosquitoes, the overnight release-recapture method or the recruitment of wild mosquitoes was used. With the release-recapture method, laboratory-reared mosquitoes were released into the experimental enclosure and later captured to record their position and status. The recruitment of wild mosquitoes, conversely, involved the capture of mosquitoes that were not raised in a controlled setting.

**Table.3:** Characteristics of included interventions

References	Study area	Research design(s)	Population description	Treatment sample size	Insecticide(s) used	Application method	Window screen or eave use
Barreaux et al. [19]	M’be, Côte d'Ivoire	Release-recapture, natural mosquito recruitment, experimental hut studies, and case–control studies	*Anopheles gambiae *sensu lato mosquitoes. Human participants identified as ‘sleepers’ and > 18 year old	Six replicates of the treatment and sleeper combination. Calculation was based on the power package in R to determine the # needed to find a 5% statistical significance at a 70–80% power calculation	Beta-cyfluthrin	Eave tube plastic inserts were machine-treated using a wettable powder formulation of the insecticide	Eaves
Barreaux et al. [12]	M'be, Côte d'Ivoire	Release-recapture, experimental hut studies, and case–control studies	*Anopheles gambiae *sensu lato mosquitoes derived from M'be and Bouake found in Cote d'Ivoire, which have been described as highly resistant to pyrethroids; also included human volunteers in experimental huts	Studies performed over 2 nights had around 90–100 female mosquitoes with 10 replicates of each treatment and 20 releases in total	Beta-cyfluthrin	Eave tube plastic inserts were machine-treated using a wettable powder formulation of the insecticide	Eaves
Chinula et al. [17]	Luangwa District, Lusaka, Zambia in Chisobe Village	Release-recapture, experimental hut studies, and case–control studies	*Anopheles gambiae* complex which partly consisted of *Anopheles arabiensis*. Also captured *Anopheles funestus* but were low in number at the end of the study. Human sleepers were all male (8 men)	2884 total *Anopheles gambiae* complex and 333 *Anopheles funestus* that dropped to fewer than 10 at end of the study. Two replicates of 16 piece WSEBs. PM dosage was 2 × more than IRS recommended concentration. BA dosage was also 14 × higher than lambda-cyhalothrin recommendation because of calculations from micro-encapsulated PM	Pirimiphos-methyl (PM)	Window screens and eaves were treated with micro-encapsulated PM	Window screens and eaves (WSEBs)
Gouissi et al. [15]	Aguégués, Benin	Case–control study	Mosquito species unidentified. Parasite identified as *P. falciparum.* 320 children as treatment and 311 children as control aged 6–59 months	Treatment installed in 70 dwellings with 320 total children. Control group consisted of 311 children	Permethrin	Researchers did not personally apply insecticide to netting. Researchers purchased Olyset Nets that were pre-impregnated with insecticide	Window screens and eaves
Kampango et al. [20]	Furvela village, Mozambique	RCT	*Anopheles funestus* and *Anopheles gambiae s. l.* mosquitoes. Houses with at least two people sleeping in each house. No other information on human volunteers	11,362 mosquitoes collected in total: 9692 *An. funestus* and 1670 *An gambiae s.l.* 16 total houses were used in the experiment	Deltamethrin and Fendozin (made of alphacypermethrin)	Deltamethrin was incorporated into the yarn fibres used to cover eaves. Fendozin was purchased as a packaged mosquito net pre-impregnated with insecticide	Eaves
Menger et al. [21]	Kigoche village, Kenya	RCT	*Anopheles coluzzii* mosquitoes used. 8 male volunteers slept in houses, one person/house. These were lab-reared but originally collected in Suakoko, Liberia	1791 mosquitoes caught in the houses. 1724 (96.3% were *Anophelines* and 67 (3.7%) were *Culicines*. 8 traditional houses with mud-walls	Delta-undecalactone	Netting was impregnated with the microencapsulated form of the insecticide. The microcapsules were created using an oil-in-water emulsion technique	Eaves
Menger et al. [22]	Kigoche village, Kenya	RCT	*Anopheles* mosquitoes were collected as well as *Culicine* mosquitoes. Male volunteers 18–28 years old (one person/house)	– In experiment 1, indoors: 7305 mosquitoes in total: 96% female and 4% male. 4496 (62%) were *Anophelines*, the rest (2809; 38%) were *Culicines*. Outdoors: 5180 caught; 97% female, 3% male. 31% were *Anophelines*, 61% *Culcines*	p-Menthane-3,8-diol (PMD)	Insecticide was purchased through a commercial repellent (Citriodiol) and sprayed on netting that was placed in eaves	Eaves
				– In experiment 2, indoors: 4137 caught (96% female, 4% male). 3266 (79%) were *Anophelines*, and 871 (21%) were *Culicines.* Outdoors: 7471 caught (88% female, 12% male). 35% *Anophelines*, 65% *Culicines*			
Mmbando et al. [18]	Ifakara Health Institute, Tanzania	Experimental huts, release-recapture, and case–control studies	Female *Anopheles arabiensis* mosquitoes. Adult volunteers, one per hut	Two huts with 500 reared *Anopheles arabiensis* deployed throughout 122 nights mosquitoes released in three cases (containing 167, 167, 166 mosquitoes each)	Transfluthrin	Eave ribbons were washed with liquid detergent (Axion) and soaked with transfluthrin	Eaves
Mmbando et al. [10]	Ifakara Health Institute, Tanzania (semi-field) and Lupiro village, Tanzania (field)	Experimental huts, release-recapture, and case–control studies	Female *An. arabiensis* mosquitoes. Two adult male volunteers	75 nights and 500 newly-reared *Anopheles arabiensis* mosquitoes every night	Transfluthrin	Eave ribbons were washed with liquid detergent (Axion) and soaked with transfluthrin	Eaves
Mwanga et al. [11]	Ifakara Health Institute, Tanzania	Experimental huts, release-recapture, and case–control studies	Laboratory-reared female *An. arabiensis* mosquitoes. Adult male human volunteers, one per hut	5 huts with 1000 *Anopheles arabiensis* mosquitoes released every night	Transfluthrin	Eave ribbons were washed with liquid detergent (Axion) and soaked with transfluthrin	Eaves
Oumbouke et al. [16]	M'be, Côte d'Ivoire	Experimental huts, release-recapture, and case–control studies	*Anopheles gambiae s.l.* mosquitoes, predominantly *Anopheles coluzzi* collected from M'be with high levels of pyrethroid and carbamate resistance levels and lab-reared. Two volunteer hut sleepers were adults who were not given prophylaxis	100 mosquitoes were released into each house	Pyrimiphos methyl, azamethiphos, *beauveria bassiana*, bendiocarb, bifenthrin, orthoboric acid, beta-cyfluthrin WP, deltamethrin, pyrethrin, butoxide, permethrin, and carbaryl	Powdered insecticides were applied to eave tube inserts placed into PVC tubes	Eaves
Snetselaar et al. [13]	Mbita Point, Kenya	Experimental houses, release-recapture, and case–control studies	*A. gambiae s.s.* and *Anopheles arabiensis* mosquitoes colonized from the Mbita area. Adult volunteers, one per hut	Two houses. 200 host-seeking female mosquitoes released outside the houses each night per house	Bendiocarb and deltamethrin	Eave tube inserts were treated with an electrostatic netting and. fluorescent or insecticide powder	Eaves
Sternberg et al. [14]	Ifakara Health Institute, Tanzania	Experimental huts, release-recapture, and case–control studies	*Anopheles arabiensis* mosquitoes from a Sagamaganga colony (nearby village) and raised in Ifakara Health Institute; adult female mosquitoes. Six human volunteers	Six huts and 200 mosquitoes released outside huts	PermaNet (treated with deltamethrin) and, bendiocarb (both wet and dry)	Netting was treated with insecticide and placed into eaves	Screening and eaves

Most studies (n = 10) reported on treated eave inserts, while three studies investigated both window screens and eaves. All data were collected in African countries: four studies took place in Tanzania, whereas three articles were aggregated in Côte d’Ivoire and three in Kenya. All other studies produced results from Mozambique, Bénin, and Zambia.

All reports (n = 13) indicated that their studies included human volunteers. Of the 13 articles, eight involved adult participants, one involved children, and four did not identify participant ages. Five studies comprised all male volunteers and the eight remaining studies did not provide information on the gender of their volunteers. Insect samples ranged from 90 to 11,362 released or collected mosquitoes. Twelve studies re-evaluated the intervention at least twice, while one design did not clearly state whether treatment replication took place (Table 2).

### Biting incidence and protective efficacy

Five of the 13 studies indicated the intervention effects on mosquito-biting incidence and protective efficacy (Table 4). Three of these reports utilized transfluthrin insecticide against mosquito vectors, of which one study found that eave ribbons treated with insecticide concentrations of 0.2% led to 100% reductions in indoor and outdoor biting with zero average nightly mosquito catches (95% CI 0.00–0.00; p < 0.001) [10]. In this report, volunteers assigned to houses with eaves covered in 10 times less transfluthrin (0.02%) still experienced 77.2% protection indoors with three average nightly catches (95% CI 0.32–5.18; p < 0.001) and 56.2% protection outdoors with 46 average nightly catches (95% CI 23.5–68.6; p < 0.001).

**Table.4:** Outcomes of included interventions

Reference	Intervention	Outcomes	Methods of measurement	Findings	Study limitations
Barreaux et al. [19]	– Two huts with insecticide-treated inserts (In2Care tubes) placed inside of eaves or an untreated insert placed into the eaves – All participants slept under a LLIN net and windows were open – All mosquitoes that entered were collected the next day. Rotated treated and untreated inserts between both huts for 24 nights	Number of mosquitoes that enter the household and mortality differences between huts	Linear mixed model to assess the number of captured mosquitoes with the insert considered as an independent variable	– Inserts treated with insecticide resulted in 46% fewer *Anopheles gambiae* mosquitoes entering the hut vs those that were untreated – Treated inserts also led to a 64% reduction in blood-feeding by mosquitoes that did make it through – Mosquitoes that entered households protected by treated eaves died more than the other hut, but this rate was not statistically significant – A statistical difference was found between nights of capture	– Used experimental houses. Released mosquitoes were reared and not wild mosquitoes which may introduce some confounding in behavior – Insecticide treated eaves may wither in potency over time due to collection of dust or nature of chemical – Mosquitoes used were only 4–5 days old, female, non-blood fed and were reared from larvae collected in the field. May have differed from other mosquitoes
Barreaux et al. [12]	Two huts, one with insecticide treated eaves (using the In2Care tool that the WHO recently finished testing in 2019) and closed windows and the other with nontreated, open windows. Monitored mosquitoes for 2 or 4 days	Number of mosquitoes that entered the hut and the mortality between the huts	Linear mixed models	– 0–0.4% of mosquitoes entered through the insecticide-treated eaves with closed windows – Around 50–80% of mosquitoes entered the control hut (untreated eaves and open windows) – Treatment hut had 25% more deaths per day compared to the control hut with 2–4% deaths per day	– Used experimental houses – Released mosquitoes were reared and not wild mosquitoes which may introduce some confoundings in behavior and wither in potency due to collection of dust
Chinula et al. [17]	– WHO wire balls with insecticidal efficacy were placed on netting each having polyacrylate-binding agents (BA) or not having BA – In one experiment, WSEB netting received 0, 1, or 2 g/sq m of micro-encapsulated PM – In other experiments, all netting received 1 or 2 g/sq PM with BA or without BA – Netting was wrapped around wire balls and mosquitoes were exposed to them for 3 min. Tested after 0, 5, 10, 15, 20 washes to see mosquito mortality	Insecticidal capabilities measured through mortality of mosquitoes	General linear mixed modeling treating experimental huts as random effects and mortality as a dependent variable	– WSEBs with PM and BA produced results similar to LLINs–killed mosquitoes at or greater than 94% after 20 total washes. This was the same result when the PM and IRS were used together. This treatment mechanism could cause WSEBs to last for years. Without the BA treatment, all mosquitoes died at 0 washes but after 10 washes, only 10% of mosquitoes died – WSEBs without PM had fewer than 5% dying consistently throughout 0, 5, 10, 15, and 20 washes with or without BA	– Performed in experimental huts. Prototype design may not be practical for all household designs and should be affordable – This prototype could not be used in real houses and only investigated polyester netting – Did not test resistance to environmental exposure such as wind, rain, or sunlight – Does not provide enough information as to how to provide major scaling up of this intervention – Unable to effectively test durability
Gouissi et al. [15]	Installed PSP Olyset nets at windows and at eaves	Measured plasmodic index, gametocyte index, parasite density, fever, hemoglobin, and anemia	Statistica 6 and performed Chi-square tests	– More prevalent cases of anemia in the control zones compared to the treatment zones (not significant). Gametocyte index did not change – Parasite density was 2 × lower in areas within the treatment zone Risk of fever was similar between the two groups	– Only performed on children. No delineation between outcomes for specifically eaves vs windows. – Treatments were also applied to doors
Kampango et al. [20]	– Researchers covered gable ends with either a four-year-old bed net, untreated shade cloth, a deltamethrin-treated shade cloth, or nothing at all – Covered the gable ends with the same material during the second week and covered the eaves with the same material during the third week	Rates of mosquito entry and the efficacy of the three different materials	Rates of mosquito entry were measured using light-trap collection. The material’s ability to protect inhabitants was measured using incidence rate ratio calculations through Generalized Estimating Equations	– *Anopheles funestus* mosquitoes did not differentially enter households that were protected using deltamethrin-treated shade cloths compared to those that were untreated completely (no shade cloth) – *Anopheles gambiae* mosquitoes did differentially enter untreated houses compared to deltamethrin-treated houses. The latter had statistically lower rates of mosquito entry by 76% – Households protected with bednetting encountered 84% *Anopheles gambiae s.l.* entry and 61.3% *Anopheles funestus* entry – Households protected with untreated shade cloth experienced 70% fewer *Anopheles funestus* entry and 69% fewer *Anopheles gambiae* entry	– Small sample size due to being a pilot study – Assumes that houses do not have any other openings. Resistance status of *An. gambiae* mosquitoes was not known
Menger et al. [21]	– Researchers used an attractant made of ammonia, lactic acid, tetradecanoic acid, and other compounds found in human skin (pull). – They also used a repellent called delta-undecalactone and added it to cotton netting placed on eaves (push). – Treated and untreated fabrics were used eight times with four replicates/day individually over 2 days. – Four interventions used included a control, a treated push fabric in eaves, an untreated pull fabric in eaves, and a treated push–pull intervention in eaves	Rates of mosquito entry and mosquito landing on fabric	SPSS and a Shapiro–Wilk test for normality. CDC trap catches and *t*-tests were used for determining house entry and significant drops in mosquito entry. GLM with Dunnet's post-hoc test was used to find the mean trap catches of the four interventions	With the repellent, mosquito entry dropped by over 50% and CDC traps caught several mosquitoes. Modeling suggests that adding the push–pull system to current malaria interventions would help to reduce mosquito biting rate by 20 × even in cases of insecticidal resistance	– Only performed on male volunteers with small sample size. Mosquitoes were lab-reared. The mathematical model may have failed to account for regional variation. Mathematical model did not include control groups – Effects of washing materials were not thoroughly considered in the study
Menger et al. [22]	Note, only experiment II in this study used repellents. Experiment II: four interventions used as push factors were: (a) eaves screened with cotton fabric impregnated with dUDL repellent; (b) control; (c) eaves treated with sprayed PMD; (d) eaves screened with untreated fabric. CDC light traps were used as the pull factor (attractant)	Mosquito entry	SPSS to find differences in mosquito house entry. Scheffe's post-hoc tests for correction	– All interventions with eave screen treatments reduced *Anopheles funestus h*ouse entry by a significant amount – Eave screening that included PMD led to a 81% drop in house entry. MM-X traps combined with eaves treated with dUDL led to a house entry drop of 35%; combined with PMD-treated eaves led to a 80% drop – PMD treated eaves had the highest drop in mosquito entry – For *Anopheles arabiensis* mosquitoes, PMD-treated eaves dropped mosquito house entry by 89%. When combined with an MM-X trap, house entry dropped by 80%, which was the only significant drop compared to the other interventions – Although PMD-eaves dropped *Culex* house entry by 84%, this was not significant	– Small sample size. Simultaneous comparison not possible in the study – Minimal analysis of the effects of resistance, and net washing
Mmbando et al. [18]	– Transfluthrin eave ribbons and odour-baited traps were tested separately and together to study their protective efficacy – Participants rested outdoors from 6:30 pm to 10:00 pm during biting activity periods and slept under a bednet from 10:00 pm to 6:30 am while light traps captured mosquitoes	Protective efficacy against biting either outdoors or indoors	Percentage of biting that took place and changes in biting patterns depending on the distance of these interventions	– The treated eave ribbon and light-traps together protected better from indoor-biting (83.4% protection) and outdoor-biting (79%) compared to solely using the traps to protect against indoor-biting (35.0%) and outdoor biting (31%). – Traps set 15 m away from huts were better at protecting individuals in combination with the treated eave ribbons against indoor-biting – Traps that were set 30 m away had better efficacy for outdoor-biting. Participants were found to have overall positive outlooks toward prototype windows, particularly the concertina windows, because of their ventilation and attraction	– Using both the treated eaves and the light-traps is complex – Study samples are limited to one type of mosquito and are based on lab-reared mosquitoes. Wild mosquitoes may behave differently. Field data lacking other factors, such as airflow, in the push–pull system. – This study could not be applied to younger populations since it only included adult volunteers
Mmbando et al. [10]	– Eave ribbons were treated with 0.02%, 0.2%, 1.5%, or 5% transfluthrin emulsion repellent and tested in two huts against 500 mosquitoes released every night. – Volunteers stayed outside each hut to collect mosquitoes from 6 to 10:00 pm and slept indoors under bed nets from 10 to 6:30 am	Indoor-biting, outdoor-biting, and mosquito survival	Prokopack aspirators to measure indoor-biting. Double-net traps to measure outdoor-biting. Survival was measured by counting the number of living mosquitoes (n = 200) in a suspended cage next to huts 24 h after exposure	– Volunteers protected by eaves with 0.2% transfluthrin experienced 99% lower indoor-biting and outdoor biting – Volunteers protected by eaves with 0.02% transfluthrin experienced 79% fewer bites indoors and 60% fewer bites outdoors – Households with no treatment resulted in 27% fewer indoor-biting and 18% greater outdoor-biting at a non-significant rate – 99.5% of mosquitoes died when exposed to treated huts – Field experiments showed that treated eaves protect at 96% indoors and 84% outdoors when tested against *Anopheles arabiensis.* These treated eaves also protect at 42% indoors and 40% outdoors when tested against *Anopheles funestus* – Mosquitoes released nightly were laboratory-reared and may not behave completely as wild mosquitoes do – Only two volunteers were used throughout the study – Colder temperatures (below room temperature) inhibit transfluthrin from vaporizing, which is essential for its repellency activities – Eave ribbons treated with transfluthrin cost $7 for construction and installation	– Mosquitoes released nightly were laboratory-reared and may not behave completely as wild mosquitoes do – Only two volunteers were used throughout the study – Colder temperatures (below room temperature) inhibit transfluthrin from vaporizing, which is essential for its repellency activities. The exact amount of transfluthrin adsorbed was not known – In the semi-field experiment, were only *An. arabiensis*; especially pertinent because the field experiment had different rates for different mosquitoes – Did not take effects of washing into consideration – Did not compare with other types of treatment for nets
Mwanga et al. [11]	– Volunteers stayed close to 5 huts protected with eave ribbons treated with 0.25 g/m2 transfluthrin at 0, 20, 40, 60, 80, and 100% coverage – All volunteers collected biting mosquitoes between 6 and 10 pm and slept under bed nets (nontreated) from 10 pm to 6 am – Mosquitoes were caged inside huts for 24 h. Huts were protected with either eave ribbons, UV-LED mosquito traps, or both, with the central hut having no insecticide-treated net to measure effects on households that do not have a net	Protection efficacy of transfluthrin eave ribbons both indoors and outdoors. Mosquito mortality against eave ribbons. Protective efficacy of combining UV-LED traps with transfluthrin eave ribbons	Collecting mosquitoes and counting remaining living ones. Also analyzed data using Generalized Linear Mixed Models	– Eave ribbons treated with transfluthrin ultimately protected volunteers at an efficacy rate of 83% indoors and 62% outdoors for volunteers who slept under a net – Volunteers who did not use nets were protected by 57% indoors and 48% outdoors – Volunteers who used nets had constant protection when eave ribbons were added and volunteers who did not use nets had increased protection when eave ribbons were added – When 80% of households were using treated eaves, the protection to non-users was at its peak – All caged mosquitoes placed inside huts with treated eave ribbons died – UV-LED traps added with eave ribbons did not improve overall protection among those who used bed nets	– Took place in semi-field settings and used lab-reared mosquitoes; may, therefore, not be representative of actual conditions – Volunteers who slept under a net slept under untreated nets, which is unrealistic since most distributed nets are treated with some form of insecticide
Oumbouke et al. [16]	Eleven powder formulations from six insecticide groups (pyrethroid, carbamate, organophosphate, neonicotinoid, entomopathogenic fungus, and boric acid) were tested against pyrethroid resistant *Anopheles gambiae s.l.* mosquitoes for four weeks	The insecticide of all 11 formulations that had the best residual efficacy, mortality data over its various transient exposures to mosquitoes, and mosquito mortality from that insecticide	Measured contact with eaves using fluorescent powder	– After four weeks, mosquito mortality dropped by 25% for the majority of all 11 insecticides even though they killed mosquitoes at 45–100% during the initial 2 weeks – Beta-cyfluthrin remained potent (100% mortality) for one month – Only < 5% of mosquitoes that came into contact with untreated eaves died. About 55% of mosquitoes that came into contact with beta-cyfluthrin treated eaves died the next morning and 64% were dead after 24 h of exposure – An average of 44% of mosquitoes came into contact with the treated eaves and 75% of mosquitoes passed through open, untreated eave tubes to enter households	– Limited in the number of insecticides this study tested and took place in experimental huts that may not represent real conditions – Mosquitoes were lab-reared – Only two volunteers were used in the experimental huts, which is a considerably small sample size. – Only *Anopheles gambiae* mosquitoes used; may have different results with different mosquito types – Did not evaluate long-term properties of all insecticides, and was not able to entirely identify what made beta-cyfluthrin the most successful
Snetselaar et al. [13]	– In two houses, eave tubes treated with bendiocarb or deltamethrin were implanted to test against *Anopheles gambiae* and *Anopheles arabiensis* mosquitoes through 3 min bioassays – Same experiment took place with untreated eave tubes (open)	The number of mosquitoes that enter each household (recapture)	Collected using record sheets and calculated by counting the number of mosquitoes retrieved. Normality was measured using the Shapiro–Wilk test and Levene's test with Mann–Whitney *U* Test and Bonferroni correction	– Households protected with bendiocarb-treated eaves had a recapture percentage of 21% on average for both *Anopheles gambiae s.s.* and *Anopheles arabiensis* mosquitoes – Those treated with deltamethrin had a recapture percentage of 39% on average for *Anopheles gambiae* mosquitoes and 22% for *Anopheles arabiensis* mosquitoes – Untreated eaves had 71% and 54% recapture rates for households only protected with fluorescent dye powder for *Anopheles gambiae* mosquitoes and 46% and 25% for *Anopheles arabiensis* mosquitoes	Was conducted in semi-field conditions with lab-reared mosquitoes. Assessed effects over several weeks, but longer term effects not evaluated in the study
Sternberg et al. [14]	– Eave tubes of various heights, diameters, angles were treated with different ingredients (bendiocarb, LLIN fabric, and fungus) applied on netting – These were tested on compartments made up of a hut and one sleeper, against open eaves, and again in a larger compartment that consisted of a mosquito population, vegetation, 2+ houses, and cattle sheds meant to represent a village. There, they brought in LLINs and then brought in eave tubes	Mortality rates between applied active ingredients. Recapture rates from treated netting. Larval densities based on LLIN introduction and larval densities based on installation of eave tubes and screening	Generalized linear modeling using quasi binomial error distributions	– Bendiocarb and LLIN netting had equal effects on mosquito mortality rates. Deltamethrin and bendiocarb applied to eaves led to 99–100% mortality of all *A. gambiae s.s.* mosquitoes, whereas 39% of *A. arabiensis* mosquitoes died under bendiocarb treatment – Treated netting resulted in 50–70% lower recapture rates compared to controls. Closed eaves (specifically ones treated with bendiocarb netting) performed similar to LLIN and open eaves together – Within the constructed village, LLINs produced a 60% drop in larval densities and 85% drop in indoor mosquitoes	– Only performed using adult human volunteers

Mmbando et al*. *indicated that eaves treated with transfluthrin afforded 96% indoor and 84% outdoor protection against *Anopheles arabiensis* [10]*.* Drastically lower, though, were the treatment results on *Anopheles funestus,* as volunteers experienced only 42% protection indoors and 40% outdoor protection [10]. As a form of community defence, one study reported that if 80% of households installed transfluthrin-eaves, an entire community would experience a state of herd immunity [8].

Mwanga et al. [11] found that eave ribbons treated with transfluthrin protected participants who slept under a net at 83% efficacy indoors and 62% outdoors. Conversely, volunteers who did not sleep under a net experienced 57% protection indoors and 48% outdoors against *An. arabiensis* mosquitoes [11]. Volunteers who slept under nets and with treated eaves had constant levels of protection, whereas those who did not sleep under a net experienced increased levels of protection when eave ribbons were added to the mix [11].

### Mosquito entry and density

Seven studies reported the intervention outcomes on mosquito entry and indoor density (Table 4). In Barreaux et al.’s [12] report, inserts treated with 10% beta-cyfluthrin and placed in eave tubes using the In2Care tool resulted in 0–0.4% of mosquitoes entering houses with closed windows. This method is known as the Lethal House Lure. The control hut had around 50–80% of mosquitoes entering the structure when eaves were untreated [12].

Snetselaar et al. [13] reported that directly applying bendiocarb to eaves produced an average recapture rate of 21% for both *Anopheles gambiae s.s.* (95% CI 18–25%) and *An. arabiensis* (95% CI 14–27%). Similarly, another report found that bendiocarb-eaves resulted in 50–70% lower recapture rates than their controls [14]. These treated eaves also performed as well as long-lasting insecticidal netting (LLIN) and open eaves together [14]. When Snetselaar et al. [13] experimented with deltamethrin, they found it allowed 18% more *An. gambiae s.s.* (95% CI 26–51%) and 1% more *An. arabiensis* (95% CI 18–25%) into houses compared to bendiocarb. Microscopically, Gouissi et al. [15] found that covering eaves and windows with Olyset Nets (impregnated with permethrin) reduced *Plasmodium* parasite density in participant blood by two times compared to the control.

### Mosquito mortality

In addition to outcomes related to mosquito entry behaviour, seven of the included studies generated reports based on mosquito mortality (Table 4). These interventions utilized the In2Care insert (n = 2), transfluthrin (n = 2), beta-cyfluthrin (n = 1), bendiocarb (n = 1), and pirimiphos-methyl (PM) insecticides (n = 1). Barreaux et al. [12] found that the In2Care tool differentially killed 25% more mosquitoes per day compared to the control hut in which 2–4% daily deaths occurred.

In another report, 11 insecticides were tested for their toxicity, wherein beta-cyfluthrin remained the most durable and potent of them all [16]. Beta-cyfluthrin was observed to maintain 100% mortality rates up to one month of use [16]. This team found that the insecticide killed 55% of mosquitoes that came into contact with treated eaves (an average of 44% of released mosquitoes) by the following morning; then, after 24 h of exposure, 64% of mosquitoes died, although not at a significant difference (p > 0.05). Within the untreated control hut, less than 5% of mosquitoes died that came into contact with untreated eaves [16].

Along with beta-cyfluthrin eaves, two interventions focused on mosquito mortality from transfluthrin-treated eaves. Eaves covered with transfluthrin were reported to result in high mortality rates. In one report, around 99.5% of all mosquitoes died upon exposure to houses protected by treated eaves [10]. Moreover, Mwanga et al. [11] found that, after exposing caged mosquitoes to treated eave ribbons, all mosquitoes reportedly died.

Chinula et al. [17] investigated the wash resistance of window screens and eave baffles (WSEBs) treated with pirimiphos-methyl (PM) insecticide and polyacrylate-binding agents (BA). Exposed mosquitoes died at rates equal to that of LLIN and continued to die at 94% after 20 washes (95% CI 0.74–0.98; p < 0.001) [17]. When BA was removed, all mosquitoes died after zero washes; however, after 10 washes, less than 10% of all mosquitoes died [17]. Likewise, when the PM insecticide was removed, less than 5% of mosquitoes died after 0, 5, 10, and 20 washes whether or not BA was present [17].

### Participant needs and attitudes

Only two articles discussed volunteer attitudes toward treated eaves or window screens (Table 4). Mmbando et al. [18] reported that participants had overall positive outlooks toward prototype windows because of their ventilation and attraction. A positive regard for these devices was demonstrated over 10 weeks by the doubling of male residents from four to eight in houses newly installed with prototype windows. Eventually, new inhabitants displayed housing comfort, redecorating 10 out of the 20 housing units with new curtains and floors [18]. Relative to bed nets, which can generally accommodate two people, eave ribbons treated with transfluthrin could accommodate four residents [10].

## Discussion

The evidence reviewed suggests that eave tubes infused with specific insecticides can reduce vector-biting incidence rates, mosquito entry rates and mosquito prevalence (Tables 3–4). Based on the studies reviewed here, insecticide-treated eaves coated with beta-cyfluthrin or transfluthrin can reduce vector biting by noteworthy amounts: A 64% drop in *An. gambiae s.l.* blood-feeding when treated with 10% beta-cyfluthrin; and a 100% drop in indoor and outdoor biting when treated with 0.2% transfluthrin [10, 19]. Transfluthrin appears to be one of the more effective insecticides on eave tubes for reducing mosquito biting, as at a concentration of 0.02% biting protection rates were 77.2% indoors and 56.2% outdoors [10]. Worth noting, however, is that transfluthrin-eaves appear to offer minimal to no protection against *An. funestus* mosquitoes [10]. Public health practitioners should therefore base the type of insecticide they treat eaves with on the most prevalent species of mosquito in a community.

Households fitted with beta-cyfluthrin-treated eaves might also be protected against mosquito entry. Of all the tests included with this insecticide, the one that followed the Lethal House Lure approach was the most successful. Using this system where beta-cyfluthrin-coated eaves were installed in houses, only 0–0.4% of mosquitoes entered these structures [12]. Local governments in malaria-endemic areas could consider implementing similar designs in their communities. Alternatively, applying bendiocarb to eaves resulted in a 79% drop in mosquito entry rates and could be used as a substitute for beta-cyfluthrin [13]. Alone, bendiocarb-eaves performed as well as LLIN coupled with untreated eaves, suggesting that together this intervention and LLIN may bolster preventative efforts [14].

Insecticide-treated eaves may also increase mosquito mortality. Transfluthrin was found to kill around 99.5% of mosquitoes in one report [10], while beta-cyfluthrin treatments killed 100% of mosquitoes for at least one month in another [16]. As impressive as most of these figures are, few studies investigated the long-term use of their interventions. This is concerning because durability is an important factor in cost–benefit analyses, raising logistical questions about replacing tools that have lost their effectiveness. The compounds in an insecticide are of varying levels of photosensitivity, meaning that exposure to sunlight initiates chemical breakdown [23]. For window screens coated with insecticide, the risk of sunlight exposure is high, eventually reducing insecticidal strength over time. Eaves, on the other hand, are situated in an enclosed space and experience less sunlight exposure. Thus, the insecticidal strength of treated eaves may last longer than on window screens.

Available evidence suggests that both window screens and eaves should be co-treated with BA to retain insecticidal potency after washes [16]. The data comes specifically from research done with PM insecticide and may not translate as accurately to other insecticides. Only two of the 13 included articles discussed participant attitudes toward any of these interventions, and both of these articles were from the same research team [10, 18]. The international community needs more data on community preferences for these tools in order that they are maintained when installed.

### Study strengths and limitations

The strengths of this review include its specificity and current analysis of treated eaves and window screens against *Anopheles* mosquitoes. Moreover, it provides comparisons between insecticides, detailing their effectiveness and, in many cases, against what species of mosquito. This information is critical for public health researchers or programmers aiming to install said devices in affected communities, as it will help them to tailor their interventions. In addition, the research team conducted this review during a time when malaria progression expressed stagnation, thereby enhancing this review’s timeliness by providing supplementary solutions [24]. The results of this study are consistent with other published reports that indicated reductions in mosquito density and biting due to eave closures or tube installations [25, 26].

Although specificity could be regarded as a strength, it may also be a limitation. Studies or corresponding results that tested netting without insecticide treatments, tested insecticidal resistance, or tested entomopathogenic fungi alone did not appear in this review. Similarly, many articles may have been overlooked due to the assessment of three databases. Only two reviewers, moreover, screened each paper for eligibility, which opens up this analysis to potential biases. The majority of studies were of a semi-field or entomological nature, and may not have similar results in real-life settings. Finally, none of the included articles explicitly discussed blinding of the investigators during their experiments [10–22].

## Conclusions

The implementation of insecticide-treated window screens and eaves could serve as useful tools to reduce mosquito biting incidence, mosquito entry, and increase mosquito mortality (Table 4). This rapid scoping review ties into the essential public health service of developing policy, as the review is a call to action for stakeholders, such as WHO, to encourage window screen and eave installations, as well as local public health agencies to support these efforts [27]. Specific insecticides that public health practitioners can consider include transfluthrin, beta-cyfluthrin, bendiocarb, and deltamethrin, although the method and community one works with should reflect that of the associated study design. Future research should more deeply study the sole impact of treated window screen installations, interventions for *An. funestus* and *An. arabiensis*, and household attitudes toward these devices.

## Data Availability

All data generated or analysed during this study are included in this published article and its supplementary information files.

## Abbreviations

WHO: World Health Organization
SES: Socioeconomic status
RCT: Randomized controlled trial
LLIN: Long-lasting insecticidal netting
WSEB: Window screens and eave baffles
PM: Pirimiphos-methyl
BA: Polyacrylate-binding agents
CASP: Critical appraisal skills programme checklist
PRISMA: Preferred reporting items for systematic reviews and meta-analyses
